# Exosomes derived from reparative M2-like macrophages prevent bone loss in murine periodontitis models via IL-10 mRNA

**DOI:** 10.1186/s12951-022-01314-y

**Published:** 2022-03-05

**Authors:** Xutao Chen, Zhuo Wan, Liu Yang, Shuang Song, Zhaoyue Fu, Kang Tang, Lihua Chen, Yingliang Song

**Affiliations:** 1 Department of Implant Dentistry, School of Stomatology, Fourth Military Medical University, Xi’an, 710032 Shaanxi China; 2 Department of Hematology, Tangdu Hospital, Fourth Military Medical University, Xi’an, 710038 Shaanxi China; 3 Department of Immunology, Fourth Military Medical University, Xi’an, 710032 Shaanxi China; 4 Department of Implant Dentistry, College of Stomatology, Xi’an Jiaotong University, Xi’an, 710004 Shaanxi China

**Keywords:** Periodontitis, M2-Exos, IL-10, Osteogenesis, Osteoclastogenesis

## Abstract

**Background:**

Periodontitis is characterized by progressive inflammation and alveolar bone loss resulting in tooth loss finally. Macrophages including pro-inflammatory M1-like macrophages and reparative M2-like macrophages play a vital role in inflammation and tissue homeostasis in periodontitis. Among them, reparative M2-like macrophages have been shown to promote tissue repair and prevent bone loss. However, the mechanism of reparative M2 macrophages-induced osteoprotective effect remains elusive.

**Results:**

Exosomes from reparative M2-like macrophages (M2-Exos) were isolated and identified successfully. M2-Exos could promote bone marrow stromal cells (BMSCs) osteogenic differentiation while suppressing bone marrow derived macrophage (BMDM) osteoclast formation, and prohibit pathological alveolar bone resorption because of the intercellular communication via exosomes. High expression level of IL-10 mRNA was detected not only in reparative M2-like macrophages but also in M2-Exos. Meanwhile, IL-10 expression level in BMSCs or BMDM was also upregulated significantly after co-culturing with M2-Exos in a concentration-dependent manner. In vitro, recombinant IL-10 proteins had the ability to selectively promote osteogenic differentiation of BMSCs and hinder osteoclast differentiation of BMDM. Moreover, after treatment with M2-Exos and IL-10R antibody together, the capacity of promoting osteogenesis and suppressing osteoclastogenesis of M2-Exos was significantly reversed. In vivo experiments further showed that M2-Exos reduced alveolar bone resorption in mice with periodontitis via IL-10/IL-10R pathway.

**Conclusion:**

In conclusion, our results demonstrate that the reparative M2-like macrophages could promote osteogenesis while inhibiting osteoclastogenesis in vitro as well as protect alveolar bone against resorption in vivo significantly. M2-Exos could upregulate the IL-10 cytokines expression of BMSCs and BMDM via delivering exosomal IL-10 mRNA to cells directly, leading to activation of the cellular IL-10/IL-10R pathway to regulate cells differentiation and bone metabolism. These results might partly account for the mechanism of osteoprotective effect of reparative M2-like macrophages and provide a novel perspective and a potential therapeutic approach on improving alveolar resorption by M2-Exos.

**Graphical Abstract:**

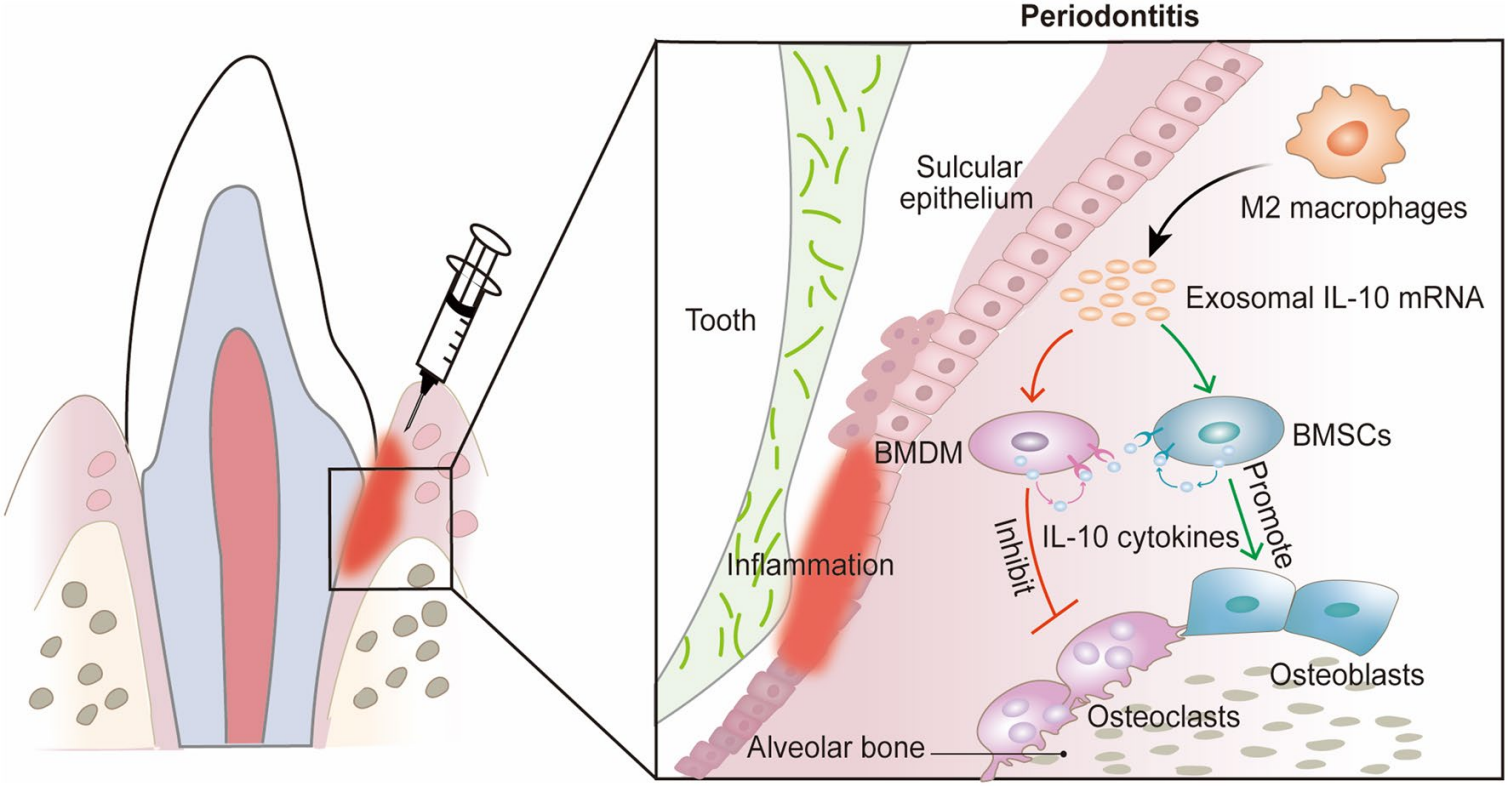

**Supplementary Information:**

The online version contains supplementary material available at 10.1186/s12951-022-01314-y.

## Background

Periodontitis, a high-incidence oral disease, is a significant public health concern affecting a vast majority of the population. Periodontitis is characterized by progressive local inflammation and loss of the alveolar bone resulting in masticatory ability insufficiency eventually. The loss of alveolar bone is closely related to the disruption of bone homeostasis regulated by osteogenesis and osteoclastogenesis. Therefore, it is very critical for periodontists to figure out the disorders of bone homeostasis to inhibit bone resorption and promote alveolar bone regeneration. As is well known, the immune system has a great effect on bone homeostasis. In the course of periodontitis development, massive immune cells infiltrate the periodontium, containing macrophages [1] which encounter immunostimulatory compounds derived from invading pathogens in the early phase of inflammation and participate in bone metabolism balance in periodontitis.

Macrophages as dynamic cells participating in induction and resolution of inflammation [2] exhibit a significant degree of plasticity and heterogeneity through the function and biology, such as pro-inflammatory M1-like macrophages at one extreme and reparative M2-like macrophages at the other extreme [3, 4]. Pro-inflammatory M1-like macrophages strongly express pro-inflammatory cytokines including IL-1β, IL-6 and TNF-α, which initiate the upregulation of destruction mediators in primary tissue and lead to bone resorption [5, 6]. Conversely, reparative M2-like macrophages secret anti-inflammatory cytokines such as TGF-β and IL-10 to suppress inflammation and maintain immunologic homeostasis [7, 8]. It is well documented that, reparative M2-like macrophages are involved in tissue repair [9] and play a pivotal role in osseous remodeling as mediators of inflammatory and immune responses [10]. The upregulated TGF-β, IL-4 and IL-10 of reparative M2-like macrophages have been supposed as major factors for bone remodeling [10–12]. Bone remodeling is a dynamic balance between osteogenesis and osteoclastogenesis. Accumulating evidence has shown that reparative M2-like macrophages can promote osteogenesis in MSCs while suppressing the early formation of osteoclasts via juxtacrine and paracrine signaling. Yet the exact mechanism of osteoprotective effects induced by reparative M2-like macrophages remains to be elucidated.

Recent studies have demonstrated that macrophages can regulate the vital biological processes of neighboring or distant cells by secreting exosomes [13]. Exosomes, a type of extracellular vesicle with diameters from 30 to 150 nm [14], are secreted by almost all types of cells under physiological and pathological conditions. Until 1980s, exosomes were found and reported for involving in cell activity, and then much attention has been paid [12, 15]. Now, they have been verified to participate in cell-to-cell communication by intercellular exchange of signal and nutrition, such as protein, nucleic acids and lipid, which hence is associated with mediating the function of targeted cells [16, 17]. Because of the signals and nutrients delivered by exosomes, macrophages are broadly involved in a number of physiological processes and pathological diseases such as cancer [18], inflammation [19] and tissue repair [20]. In addition, macrophages exosomes also participate in bone remodeling [21]. However, the exosomes derived from different types of macrophages have been shown to exert different functional effects on osteogenesis [22, 23]. Among them, exosomes from reparative M2-like macrophages (M2-Exos) are gradually regarded as a positive mediator on osteogenesis [24].

Current standard treatment includes basic periodontal therapy and surgical periodontal treatment with systemic or local concomitant antibiotherapy [25], whereas the treatment for periodontitis was far from satisfactory. Many studies have shown that promoting macrophage polarization to the reparative M2 phenotype significantly inhibits bone resorption due to periodontitis [26]. Accordingly, we aim to investigate the osteoprotective mechanism of reparative M2-like macrophages and develop a novel method for promoting bone formation and decreasing bone resorption during periodontitis. Our recent study demonstrated that M2-Exos could promote osteogenesis and suppressed osteoclastogenesis. Moreover, IL-10 expression was upregulated remarkably in both bone marrow stromal cells (BMSCs) and bone marrow derived macrophage (BMDM) after incubating with M2-Exos. IL-10 is highly expressed in reparative M2-like macrophages and described as a cytokine synthesis inhibitory factor, which has been extensively shown to lower the synthesis of pro-inflammatory cytokines. Furthermore, IL-10 is also considered to be a crucial regulator for bone homeostasis and inflammatory conditions [27], playing a prominent role in regulating osteoblast/osteoclast differentiation and function. Therefore, we hypothesized that reparative M2-like macrophages may exert its osteoprotective effect through up-regulating the IL-10 expression in both BMSCs and BMDM via transporting exosomal IL-10 mRNA. Here, we demonstrate the effect of M2-Exos on inducing BMSCs toward the osteoblasts and inhibiting BMDM toward the osteoclasts via exosomal IL-10 mRNA, as well as inhibiting bone resorption during periodontitis.

## Results

### Isolation and characterization of M2-exos

**Fig. 1:**
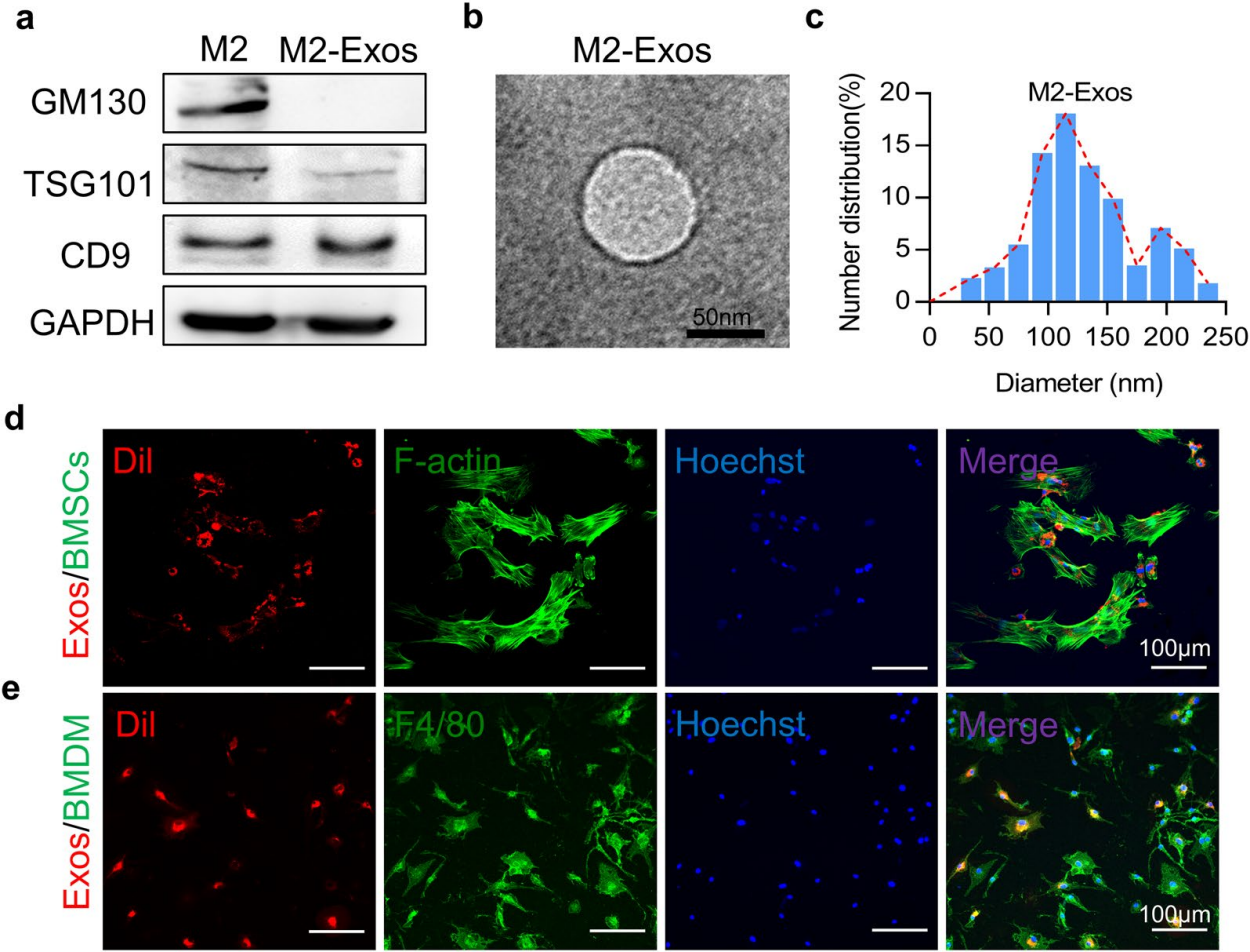
Characterization of isolated exosomes. **a** Western blotting analysis revealed the proteins of TSG101 and CD9 were enriched in exosomes. **b** Scanning electron microscopy was used to identify the vesicles isolated from reparative M2-like macrophages. **c** Particle size distribution of the exosomes secreted by reparative M2-like macrophages were investigated by dynamic light scattering (DLS) analysis. Exosomes derived from reparative M2-like macrophages was labeled with red fluorescence DiI and co-cultured with BMSCs (**d**) and BMDM (**e**) respectively, red fluorescence represents exosomes in BMSCs or BMDM

To determine the feature of exosomes from the reparative M2-like macrophages, we purified the exosomes from the serum-free culture supernatants of reparative M2-like macrophages by ultracentrifugation. M2-Exos were measured to be 30–150 nm in diameter, as demonstrated by both TEM and NLS analysis (Fig. 1b, c). As shown in Fig. 1b, the morphology of M2-Exos under TEM was round or round-like vesicle with a bilayer lipid membrane. Western blot further showed the isolated vesicles expressed the exosomal markers of CD9 and TSG101 without the cell-specific marker GM130 (Fig. 1a). Subsequently, our findings revealed that the M2-Exos can be endocytosed by BMSCs and BMDM, respectively. The M2-Exos labeled with the fluorescent dye DiI were incubated with BMSCs (Additional file 1: Fig. S1a, b) or BMDM (Additional file 1: Fig. S2) for 3 h, and then images showed DiI-labeled M2-Exos were localized in the cytosol (Fig. 1d, e). Collectively, exosomes were successfully extracted from reparative M2-like macrophages and efficiently taken up by BMSCs or BMDM.

### M2-Exos regulate the differentiation of BMSCs and BMDM

**Fig. 2:**
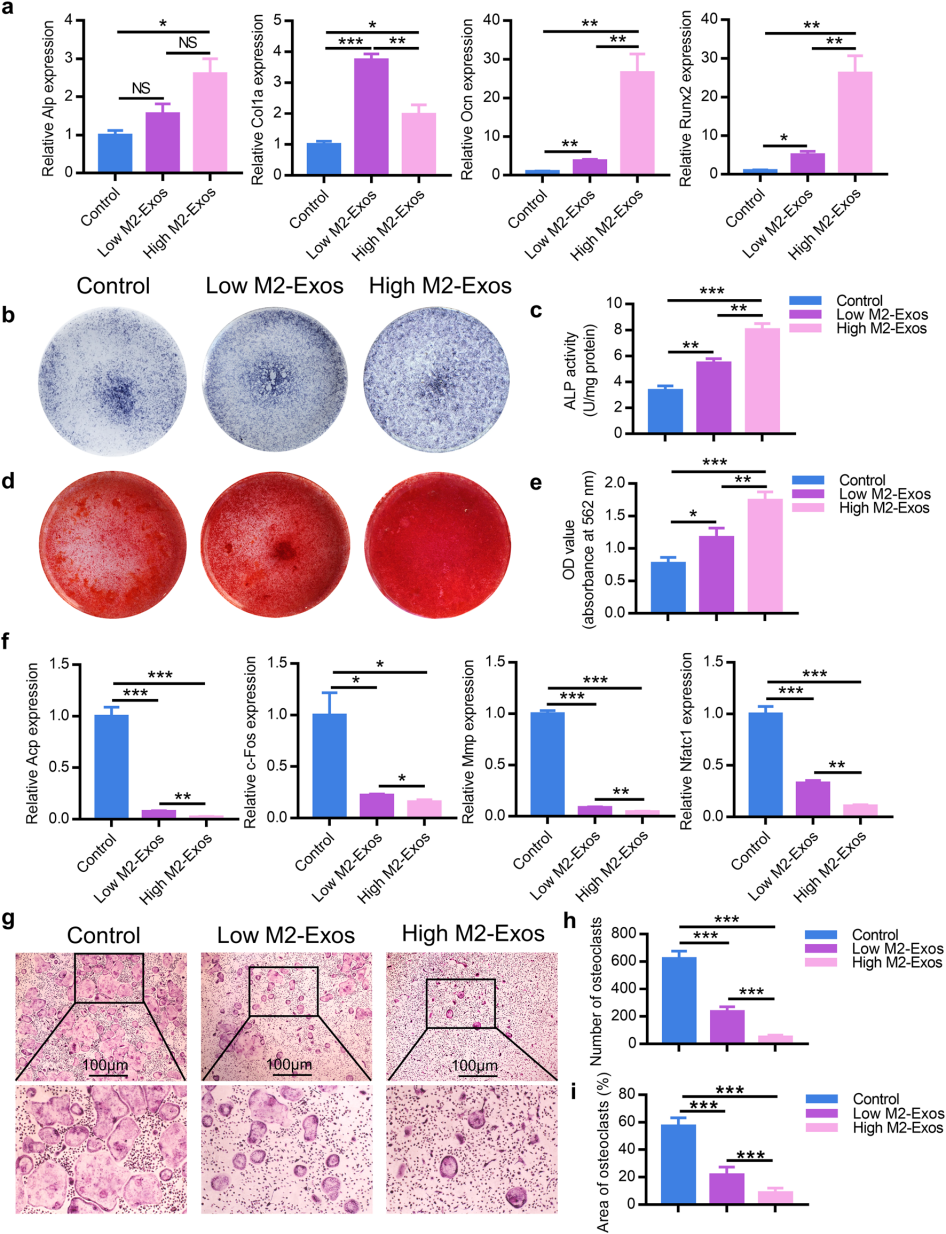
M2-Exos promote osteogenesis and suppress osteoclastogenesis in vitro. **a** Osteogenesis-related genes were upregulated in M2-Exos-treated BMSCs quantified by RT-PCR analysis. **b** ALP staining in BMSCs following treated by an equal volume of PBS, 50 μg/ml M2-Exos, or 100 μg/ml M2-Exos for 14 d. **c** ALP activity was examined via an ALP assay kit. **d** Alizarin red-mediated calcium staining in BMSCs following treated by an equal volume of PBS, 50 μg/ml M2-Exos, or 100 μg/ml M2-Exos for 21 d. **e** Alizarin red staining was extracted by cetylpyridinium chloride and quantified by spectrophotometer. **f** Osteoclastogenesis-related genes were inhibited in BMDM induced osteoclasts treated with M2-Exos by RT-PCR analysis. **g** TRAP staining was performed in osteoclasts induced by BMDM treated by an equal volume of PBS, 50 μg/ml M2-Exos, or 100 μg/ml M2-Exos for 7 d. Thse number (**h**) and area (**i**) of TRAP-positive cells was calculated. Data are mean ± SEM of triplicate experiments. *p < 0.05, **p < 0.01, ***p < 0.001

We next investigated how M2-Exos exerted osteoprotective effect by modulating differentiation of BMSCs and BMDM in vitro. BMSCs were treated with an equal volume of PBS, 50 μg/ml M2-Exos, or 100 μg/ml M2-Exos released into osteoinductive medium respectively. Expression of Alp, Col1a, Runx2 and Ocn were then ascertained by RT-qPCR 3 d post incubation. As compared with the control group, the expression levels of these genes were significantly upregulated in the M2D-Exos group in a concentration-dependent manner (Fig. 2a). To further explore the impact of M2-Exos on osteogenesis, BMSCs were continuously cultured for 21 d. Subsequently, the ALP staining and alizarin red staining were assessed on day 14 and 21, respectively, which revealing enhanced osteogenic capability and mineral deposition in M2-Exos-treated BMSCs (Fig. 2b–e). Meanwhile, we co-cultured M2-Exos with BMDM in the same conditions as BMSCs with osteoclastic medium for 7 d. The multiple osteoclastogenic-related genes expression, including Acp, Nfatc1, c-Fos and Mmp-9, were negatively correlated with the concentration of M2-Exos (Fig. 2f), and the differentiation of osteoclasts was found to be markedly suppressed (Fig. 2g–i).

### M2-Exos decrease alveolar bone resorption in mice with periodontitis

**Fig. 3:**
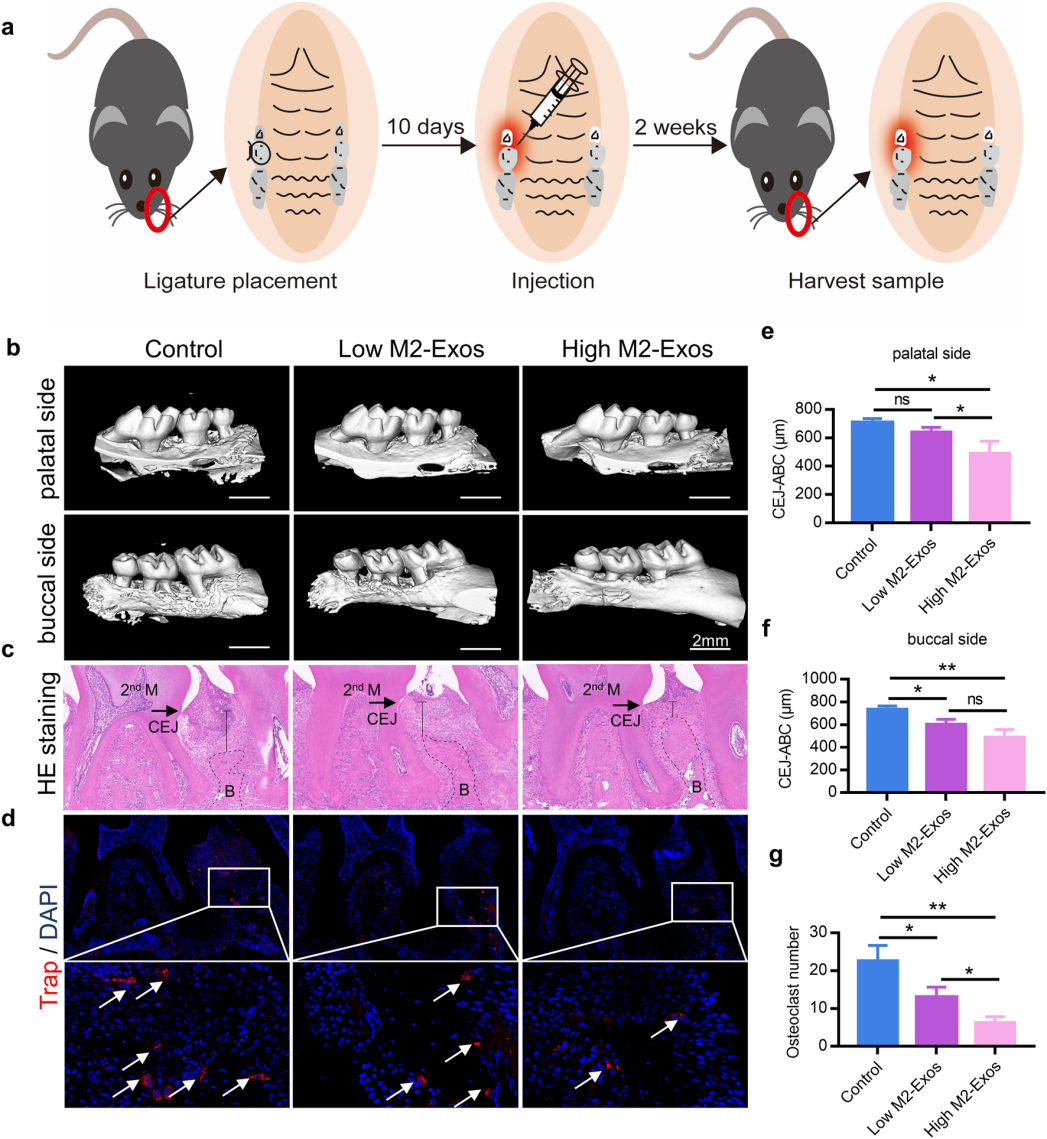
M2-Exos attenuate alveolar bone resorption in periodontitis mice. **a** Schematic diagram showing the process of construction of the periodontitis animal model and exosomes injection into mice. **b** 3D-reconstructed images produced by micro-CT were performed on maxillae of PBS, 50 μg/ml M2-Exos, or 100 μg/ml M2-Exos treated groups. The average distance from alveolar bone crest (ABC) to cement-enamel junction (CEJ) on (**e**) three palatal sides (mesial, central, and distal sites) and (**f**) three buccal sides (mesial, central, and distal sites) of left maxillary second molar was measured. Data are shown as means ± SEM. **c** H&E staining images showing the alveolar bone resorption of the periodontium from each group. **d** Confocal analysis showing TRAP-stained osteoclasts (arrows) in the sections of periodontium from each group. Blue, nuclei; red, osteoclasts. **g** The number of TRAP-stained osteoclasts was counted via microscope in each group. Data are mean ± SEM. *p < 0.05, **p < 0.01, ***p < 0.001. 2nd M, second molar; CEJ, cementum-enamel junction; B, bone

To assess the impact of M2-Exos in periodontitis mice, we used molar ligation with P. gingivalis to induce periodontitis. The ligature was tied around the maxillary left second molar for 10 d and then the mice with periodontitis were locally injected a total of 30 μl samples with PBS, 50 μg/ml M2-Exos, or 100 μg/ml M2-Exos respectively in the maxillary left buccal mucosa on days 1, 4, and 7 after ligature removal (Fig. 3a). Micro-CT examinations were used to assay the alveolar bone-resorption process of periodontitis healing. Compared with control group, the alveolar bone-resorption in M2-Exos treated mice was reduced significantly and exhibited greater osteoprotective effect in the buccal side than palatal side of alveolar bone (Fig. 3b, e and f). In addition, the samples of maxilla were harvested for H&E staining post M2-Exos treatment. H&E staining results revealed similar results as micro-CT examinations (Fig. 3c). Moreover, immunofluorescence further revealed that TRAP expression in maxillary tissue of M2-Exos group was substantially reduced compared with control mice, which demonstrated lessened osteoclastic lineage (Fig. 3d, g).

### IL-10 is involved in osteoblastic differentiation and osteoclastic inhibition

**Fig. 4:**
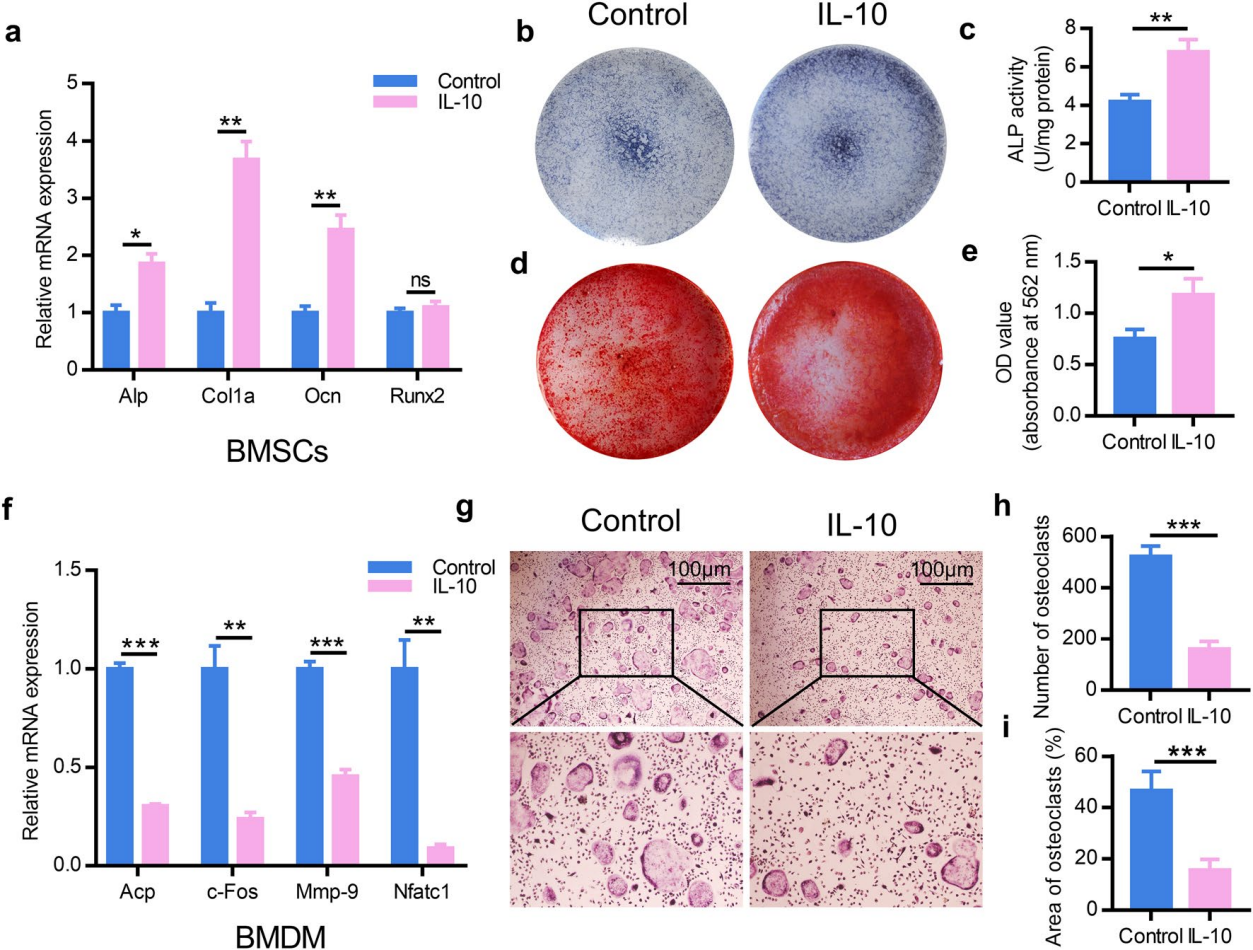
IL-10 activates osteoblast differentiation and inhibited osteoclast differentiation. **a** Osteogenesis-related genes were upregulated in BMSCs treated with IL-10 recombinant proteins quantified by RT-PCR analysis. **b** ALP staining in BMSCs following treated by IL-10 recombinant proteins for 14 d. **c** ALP activity was examined via an ALP assay kit. **d** Alizarin red-mediated calcium staining in BMSCs following treated by IL-10 recombinant proteins for 21 d. **e** Alizarin red staining was extracted by cetylpyridinium chloride and quantified by spectrophotometer. **f** Osteoclastogenesis-related genes were inhibited in BMDM induced osteoclasts treated with IL-10 by RT-PCR analysis. **g** TRAP staining was performed in osteoclasts induced by BMDM treated with IL-10 for 7 d. The number (**h**) and area (**i**) of TRAP-positive cells was calculated. Data are mean ± SEM. *p < 0.05, **p < 0.01, ***p < 0.001

Previous studies demonstrated that IL-10 was generated highly by reparative M2-like macrophages and regarded as one of markers to reparative M2-like macrophages. Next, we explored the ability of IL-10 to directly affect BMSCs or BMDM by treating cells with IL-10 recombinant proteins respectively. After incubated with IL-10 recombinant proteins, cells were harvested and the results indicated that osteogenic-related genes, including Alp, Col1a, Runx2 and Ocn, were then measured by RT-qPCR analysis, showing a marked improvement of these genes in the IL-10 group (Fig. 4a). Furthermore, the detection of alizarin red staining and extracellular matrix mineralization on day 14 and 21, respectively, revealed a clearly increased capacity of osteogenic differentiation of BMSCs in IL-10 group (Fig. 4b–e). Simultaneously, after co-culturing with IL-10 recombinant proteins, the capacity of BMDM differentiating into osteoclasts was significantly inhibited (Fig. 4f–i). Taken together, these results thus confirmed the ability of IL-10 to directly result in osteoblastic differentiation and osteoclastic inhibition.

### M2-Exos promote IL-10 cytokines expression of BMDM and BMSCs via delivering exosomal IL-10 mRNA to cells directly

**Fig. 5:**
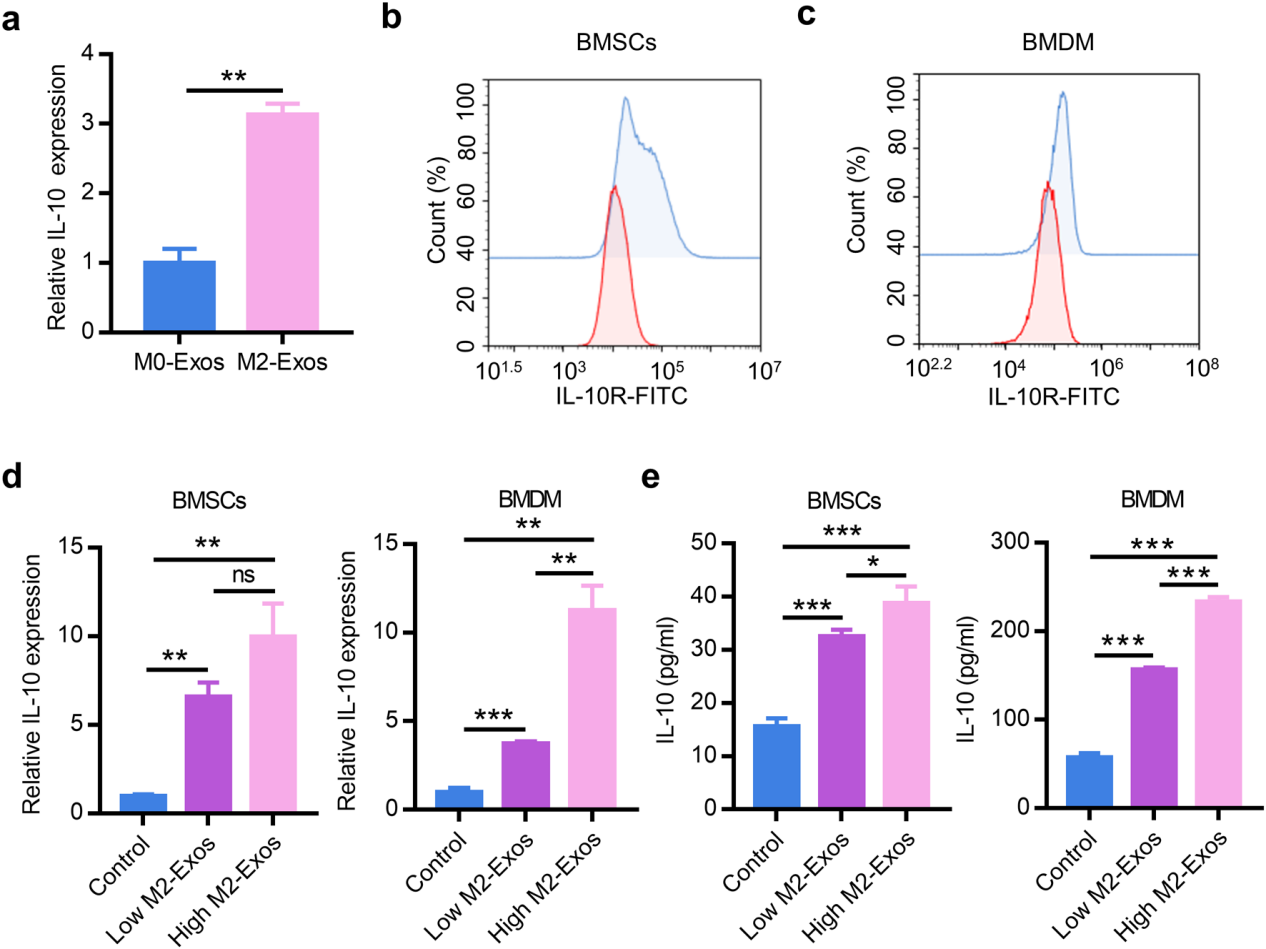
M2-Exos elevate IL-10 cytokines expression of BMDM and BMSCs via delivering exosomal IL-10 mRNA to the cells directly. **a** Total RNA was extracted from exosomes and quantified by RT-PCR analysis. Surface IL-10R expression of BMSCs (**b**) and BMDM (**c**) was monitored by flow cytometry. **d** The expression of IL-10 was measured in BMSCs or BMDM treated by PBS, 50 μg/ml M2-Exos, or 100 μg/ml M2-Exos by RT-PCR analysis. **e** IL-10 cytokines in culture supernatant of BMSCs or BMDM were measured by ELISA analysis

Considering that exosome can act as a delivery vehicle carrying signaling molecules in a paracrine manner and the high expression of IL-10 in reparative M2-like macrophages, we isolated the exosomes from M0 and reparative M2-like macrophages culture supernatants and then IL-10 mRNA was detected and found to be expressed higher in M2-Exos compared with M0-Exos (Fig. 5a). Next, cells were harvested at 6 h by co-culturing different concentrations of M2-Exos with BMSCs and BMDM, respectively. The results showed that the expression level of IL-10 mRNA was elevated significantly in both BMSCs and BMDM in a concentration-dependent manner (Fig. 5d). Moreover, with fresh medium for an additional 24 h after incubating cells and M2-Exo as above, the IL-10 cytokines in supernatants were measure via ELISA analysis. As Fig. 5e shown, IL-10 cytokines in supernatants of in BMSCs and BMDM were also remarkably increased in a concentration-dependent manner. Subsequently, further experiment confirmed that IL-10R was expressed on the membranes of BMSCs or BMDM by flow cytometry analysis (Fig. 5b, c). Thus, these results suggested that M2-Exos may deliver IL-10 mRNA to BMSCs and BMDM to promote IL-10 cytokines expression and exert the effect via IL-10/IL-10R pathway.

### M2-Exos modulate cells differentiation via IL-10/IL-10R pathway

**Fig. 6:**
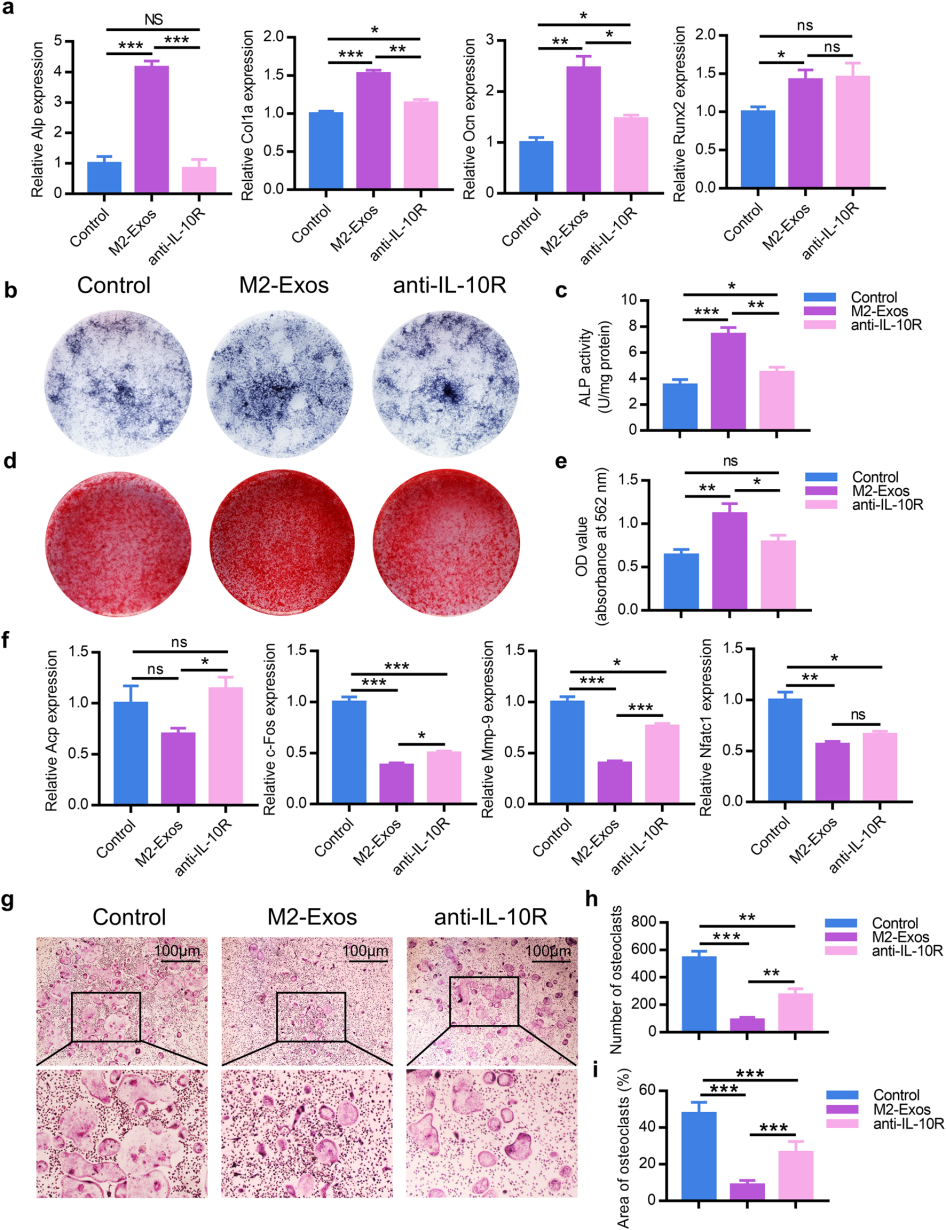
M2-Exos promote osteogenesis and suppress osteoclastogenesis via IL-10/IL-10R pathway. **a** Osteogenesis-related genes were quantified in BMSCs treated by PBS, M2-Exos, or M2-Exos with anti-IL-10R antibody by RT-PCR analysis. **b** ALP staining in BMSCs following treated by PBS, M2-Exos, or M2-Exos with anti-IL-10R antibody for 14 d. **c** ALP activity was examined via an ALP assay kit. **d** Alizarin red-mediated calcium staining in BMSCs following treated by PBS, M2-Exos, or M2-Exos with anti-IL-10R antibody for 21 d. **e** Alizarin red staining was extracted by cetylpyridinium chloride and quantified by spectrophotometer. **f** Osteoclastogenesis-related genes were measured in BMDM induced osteoclasts treated by PBS, M2-Exos, or M2-Exos with anti-IL-10R antibody by RT-PCR analysis. **g** TRAP staining was performed in osteoclasts induced by BMDM treated by PBS, M2-Exos, or M2-Exos with anti-IL-10R antibody for 7 d. The number (**h**) and area (**i**) of TRAP-positive cells was calculated. Data are mean ± SEM. *p < 0.05, **p < 0.01, ***p < 0.001

To further verify M2-Exos could regulate the differentiation of BMSCs and BMDM via IL-10, we incubated BMSCs or BMDM with an equal volume of PBS, M2-Exos and M2-Exos with anti-IL-10R antibody released into osteoinductive medium respectively. Expression of Alp, Col1a, Runx2 and Ocn were then quantified by RT-qPCR 3 d post incubation. As compared with the M2-Exos group, the mRNA expression levels of these genes were significantly down-regulated in the anti-IL-10R antibody group (Fig. 6a) and the capacity of osteogenic differentiation of BMSCs in anti-IL-10R antibody group was restrained (Fig. 6b–e). Instead, after co-cultured with PBS, M2-Exos and M2-Exos with anti-IL-10R antibody released into osteoclastic medium respectively for 7 d, suppressed osteoclastogenic-related genes including Acp, Nfatc1, c-Fos and Mmp-9 of BMDM in M2-Exos group were upregulated in anti-IL-10R antibody group (Fig. 6f). Moreover, the osteoclastic differentiation of BMDM was mainly reversed (Fig. 6g–i). The results above revealed that M2-Exos could regulate osteogenic differentiation of BMSCs and osteoclastic differentiation of BMDM via IL-10/IL-10R pathway.

### M2-Exos derived IL-10 reduce alveolar bone resorption in mice with periodontitis

**Fig. 7:**
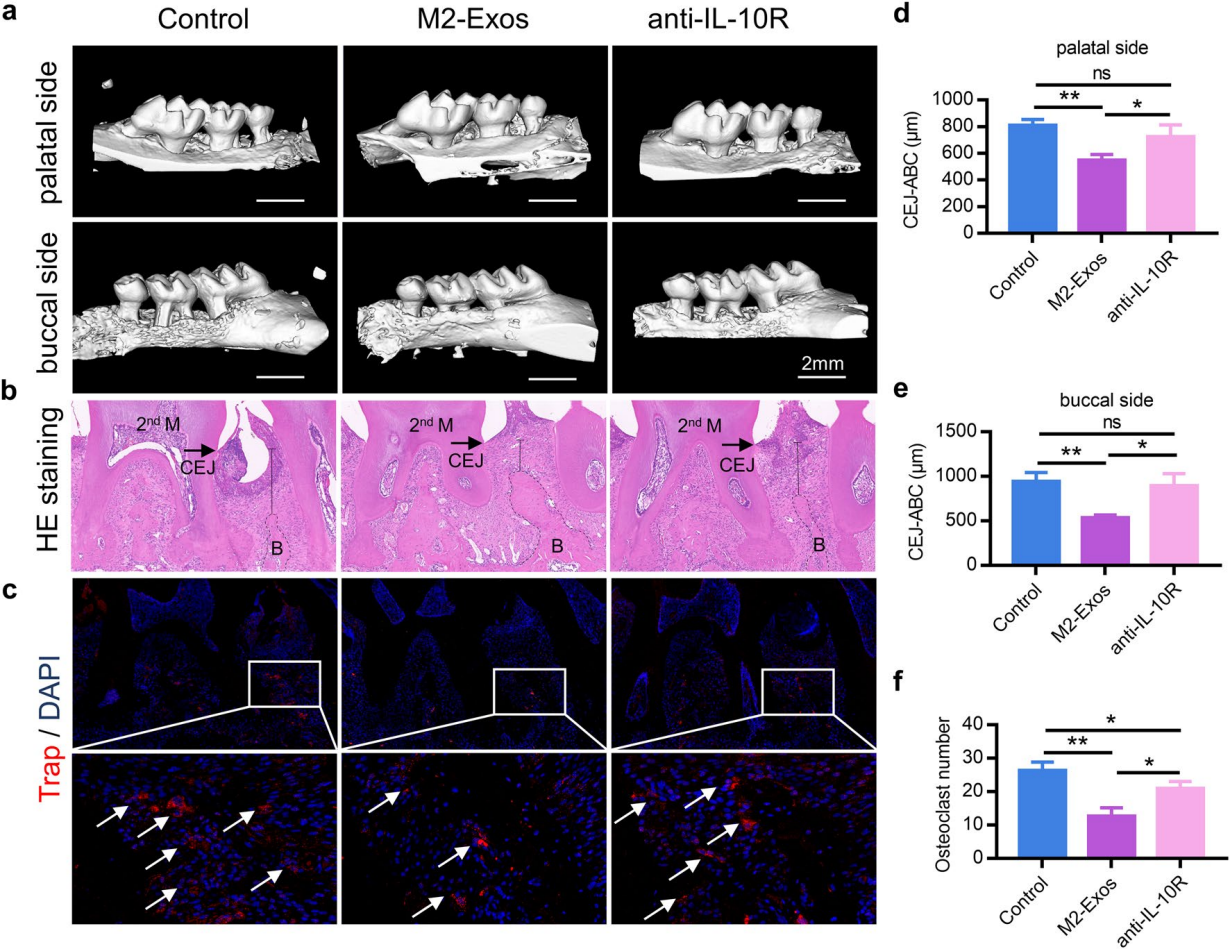
M2-Exos reduce alveolar bone resorption in periodontitis mice via IL-10/IL-10R pathway. **a** 3D-reconstructed images produced by micro-CT were performed on maxillae of PBS, M2-Exos, or M2-Exos with anti-IL-10R antibody treated groups. The average distance from alveolar bone crest (ABC) to cement-enamel junction (CEJ) on (**d**) three palatal sides (mesial, central, and distal sites) and (**e**) three buccal sides (mesial, central, and distal sites) of left maxillary second molar was measured. Data are shown as means ± SEM. **b** H&E staining images showing the alveolar bone resorption of the periodontium from each group. **c** Confocal analysis showing TRAP-stained osteoclasts (arrows) in the sections of periodontium from each group. Blue, nuclei; red, osteoclasts. **f** The number of TRAP-stained osteoclasts was counted via microscope in each group. Data are mean ± SEM. *p < 0.05, **p < 0.01, ***p < 0.001. 2nd M, second molar; CEJ, cementum-enamel junction; B, bone

To investigate how M2-Exos decreased bone resorption caused by periodontitis, we administered PBS, M2-Exos, or M2-Exos with anti-IL-10R antibody respectively to the site of maxillary left buccal mucosa in murine models on days 1, 4, and 7 after ligature removal. Maxillary samples were harvested and then monitored by micro-CT, the results showed a significantly absorption of alveolar bone in PBS groups and on day 7 post ligature removal, the M2-Exos-treated mice no longer increased bone resorption suggesting bone remodeling had clearly taken place. However, when blocking the IL-10/IL-10R pathway via anti-IL-10R antibody, skeletogenesis was substantially reduced in maxillary tissue compared with M2-Exos group which suggested the positive effect of M2-Exos was significantly reversed (Fig. 7a, d and e). In addition, H&E staining further indicated reduced alveolar bone resorption for mice treated with M2-Exos group and blocked the IL-10/IL-10R pathway can impair this positive bone remodeling effect (Fig. 7b). Moreover, immunofluorescence revealed that the number of osteoclasts was decreased in M2-Exos group compared with control group. And the number of osteoclasts was increased after blocking the IL-10/IL-10R pathway with anti-IL-10R antibody (Fig. 7c, f). The results above suggested that M2-Exos could positively regulate bone remodeling via IL-10/IL-10R pathway in periodontitis.

## Discussion

The inflammatory microenvironment of periodontitis could activate osteoclast differentiation by RANKL/RANK/OPG system. In parallel, the immune cells and activated osteoclasts can secrete chemokines, such as CXCL12, to facilitate BMSCs recruitment and migration. Induction and activation of BMSCs or periodontal ligament stem cells into osteoblasts is the key for the regeneration of alveolar bone [28]. Bone homeostasis requires a balance between osteogenesis (bone formation) and osteoclastogenesis (bone resorption) [29]. However, periodontitis was reported that can suppress the alveolar bone healing via disturbing osteogenic and osteoclastic balance resulting in the loss of teeth [30]. Macrophages have been reported to be phenotypically plastic and can be activated into reparative M2-like macrophages [31], which can promote osteoblast differentiation while inhibiting osteoclast formation [32, 33]. Therefore, gaining further insights into the mechanisms of reparative M2 macrophages-induced osteoprotective effect will be of great significance for the treatment of periodontitis.

Exosomes from donor cells can modulate intercellular communication by transporting biomolecules including RNAs, proteins and lipids, and thus induce characteristics to recipient cells so that they will be moderated in the changing microenvironment [34]. Therefore, exosomes were important for maintaining normal physiological activity and impacting pathological processes. Accumulating evidence suggests that exosomes could play an essential role in the progression of bone remodeling. It was reported that MSCs-derived exosomes could induce the formation of osteogenic lineages from naive stem cells [35] and the mineralizing osteoblast could establish a positive feedback via exosomes itself thereby promoting MSCs osteogenic differentiation [36]. Moreover, studies have demonstrated that the exosomes derived from monocyte cell [37] and dendritic cell are also able to promote MSCs osteogenic differentiation significantly in vitro, which further suggest the importance of exosomes in bone metabolism.

In this study, we found that the osteogenic differentiation capacity of BMSCs was significantly enhanced after exposure to M2-Exos. Simultaneously, the osteoclast formation of BMDM was suppressed remarkably in the presence of M2-Exos in vitro. In the mouse model of periodontitis, bone tissue defects were reduced by local injection of M2-Exos in inflammatory gingival tissue around teeth and the number of TRAP-positive osteoclasts was dramatically decreased. These results were consistent with the results of experiments in vitro described above and suggested that M2-Exos could regulate the balance of bone metabolism by directly affecting both osteoblasts and osteoclasts to reduce bone loss in periodontitis. However, the mechanisms by which M2-Exos confer bone remodeling via osteoclast and osteoblast remains unclear. Previous studies have shown that reparative M2-like macrophages express IL-10 highly which is associated with alveolar bone homeostasis and participate in regulation of the inflammatory responses [27]. Marcela et al. [38] demonstrated that in the absence of IL-10, alveolar bone loss was closely related with reduced expression of osteoblastic and osteoclastic markers, independent of the effects of microbial, inflammatory or bone resorption pathways. Hence, IL-10 is considered to be a potential mediator of bone homeostasis for periodontitis. In this study, by adding recombinant protein IL-10 to the medium of BMSCs and BMDM respectively, we found osteogenesis was enhanced in BMSCs while osteoclast formation is restrained in BMDM. The results were in agreement with the previous results, which suggested that M2-Exos may exert its effects by IL-10/IL-10R pathway.

Exosomes are lipid bilayer nanovesicles that contain nucleic acids, lipids and proteins. However, exosomes have a limited capacity to deliver macromolecules, such as messenger RNAs, leading to low yields of mRNA or proteins by exosomes. For this reason, the function of mRNA in exosomes is limited. Interestingly, Chen et al. [39] showed that IL-10 was concentration-dependent in osteogenesis of BMSCs and have a dual role in osteogenesis. Low physiological concentrations of IL-10, such as 0.01 ng/ml, can promote osteogenesis in BMSCs, but with higher doses of IL-10, the ability of osteogenesis is inhibited. Hence, low delivery of exosomal IL-10 mRNA may positively regulate the bone metabolic balance. The results we found that the concentration of IL-10 cytokine in the supernatant of BMSCs after incubated with M2-Exos ranging from 0.01 to 0.1 ng/ml, which were consistent with the results mentioned above.

In the course of the present study concerning mechanisms of M2-Exos-induced osteoprotective effect, a significant increase in IL-10 mRNA expression in exosomes was detected during the polarization of M0 macrophages to reparative M2-like macrophages in this study. By co-culturing M2-Exos with BMSCs and BMDM respectively, the results indicated that IL-10 expression was elevated in BMSCs and BMDM in a concentration-dependent manner. Subsequently, we used anti–IL-10R to block IL-10/IL-10R pathway and the capacity of osteogenesis promotion and osteoclastogenesis suppression caused by M2-Exos were limited in vitro and in vivo. These results suggested that the osteoprotective effect of reparative M2-like macrophages may be mediated by exosomal delivery of IL-10 mRNA to cells, which in turn increases cellular IL-10 expression and activates IL-10/IL-10R pathway.

Although we identified exosomal IL-10 mRNA as a potential target of M2-Exos to regulate bone metabolism, there are several limitations of our study. First, the stimulatory effect of M2-Exos on osteoblasts and osteoclasts cannot be completely inhibited by blocking IL-10 signaling. Given the characteristics of exosomes delivering cargo, other biomolecules such as miRNA and proteins may be also involved in the regulation of bone remodeling, which require further research for validation. Second, in animal study, the function of M2-Exos to reduce the loss of the palatal side of the alveolar bone is limited compared with buccal side. This may be due to the thinner buccal bone and more M2-Exos enrichment because of the buccal injection site, which lead to a better therapeutic effect. In further studies, we plan to introduce slow-release carriers or bone grafting materials to further enhance the ability of M2-Exos to promote osteogenesis. Third, the ability of macrophages to yield exosomes is insufficient as required for clinical translation. Therefore, further studies about yield promotion and increasing drug loading capability of exosomes are still needed.

## Conclusion

Overall, the results presented in this study indicate that M2-Exos may exert osteoprotective effect via transporting IL-10 mRNA to BMSCs and BMDM respectively to upregulate the expression of IL-10 cytokines, which significantly results in osteogenesis promotion and osteoclastogenesis suppression in vitro and in vivo. Our results provide a new insight into the mechanism by which M2-Exos regulate the bone metabolism and the bone repair process, revealing a novel therapeutic strategy for periodontitis.

## Methods

### Cell isolation and culture

BMSCs and BMDM were harvested from long bones of sacrificed C57BL/6 (6–8 weeks of age) mice. After removing both ends of the tibiae and femur bone, the medullary cavity was rinsed with the aid of syringe under sterile conditions. Cells were harvested and resuspended into the culture flask. The culture medium consisted of α-modified Eagle’s medium-α (α-MEM, Gibco, USA), 10% fetal bovine serum (FBS, Gibco, USA) and 1% Penicillin–streptomycin (100 IU/mL penicillin and 100 µg/mL streptomycin) (Life Technologies) in a humidified 5% CO^2^ incubator. The culture medium was replaced every 48 h and the cells were passaged at 80–90% confluency. The BMSCs of 3rd generation were prepared for follow-up experiments. For BMDM culture, bone marrow cells were gathered and plated at 10-cm dish with Dulbecco’s modified Eagle’s medium (DMEM, Gibco, USA) supplemented with 10% fetal bovine serum, 1% Penicillin–streptomycin and 25 ng/ml M-CSF (Biolegend, USA). Cells were cultured for 6 d with the medium changed every 48 h. Then, 20 ng/ml IL-4 (Biolegend, USA) was administered as reparative M2-like macrophages stimulators for 24 h and used in subsequent experiments.

### Isolation of exosomes

After 24 h incubation with 20 ng/ml IL-4, induced reparative M2-like macrophages were washed with phosphate-buffered saline (PBS) for 3 times and then were cultured in exosome-free medium. After 48 h culturing, dead cells and debris from cellular supernatants were discarded by centrifuging at 3000×*g* for 15 min, followed by filtration with a 0.45 μm filter. The supernatants were harvested and underwent ultracentrifugation for 90 min at 120,000*g* at 4 °C. The precipitate was resuspended by PBS and spun for an additional 90 min at 120,000*g*. The final precipitate (exosomes) was dissolved in 500 μL of 1 × PBS and stored at − 80 °C.

### Characterization of exosomes

Transmission electron microscopy (JEM-2000EX TEM, Japan) was used to assess the morphology of purified reparative M2-like macrophages derived exosomes. Adequate exosomes solution was placed onto 200 mesh copper grids for 5 min at 37 °C and then 2% phosphotungstic acid was used to stain the copper grids. When the grids are dry, the exosomes were viewed and photographed under TEM. Collected M2-Exos were diluted to 500 ng/ml and to avoid inter-particle interaction. The size distribution of extracted exosomes was investigated by Nanoplus (Beckman Coulter, USA).

### Internalization of DiI-labeled exosomes in vitro

To determine whether M2-Exos can be uptake by BMSCs and BMDM, the exosomes were labeled by 1 μM DiI lipophilic dye (Invitrogen, USA). After incubated with DiI for 30 min, exosomes were collected via centrifugation at 120,000*g* for 90 min, followed by adding to the culture medium of BMSCs and BMDM for 6 h, respectively. Then phalloidin was added to stain F-actin in BMSCs and BMDM staining was performed using anti-F4/80. Afterward, the nuclei of treated cells were incubated with Hoechst (Invitrogen, USA) for 10 min at room temperature. The images were viewed via a fluorescence microscope (Olympus, Japan).

### Alizarin red staining

BMSCs were plated onto 12-well plates. When reaching 80–90% confluence, the osteogenic induction medium containing 10 mM b-glycerophosphate, 100 nM dexamethasone and 50 mM ascorbic acid was used to promote osteogenesis. The osteogenic induction medium was changed every 3 d. After 21 d induction, BMSCs were washed by PBS for twice. With 4% paraformaldehyde was then added to fix the cells for 15 min at room temperature. Alizarin Red S staining was performed by staining the cells with Alizarin Red S solution (Sigma-Aldrich, USA) for 20 min. Subsequently, the distilled water was used to rinse twice and the red mineralized nodules were observed and captured using an inverted microscope (Olympus, Japan). Absorbance was then measured at 562 nm.

### ALP staining and activity assay

14 d after osteogenic induction of BMSCs, a BCIP/NBT alkaline phosphatase color development kit (Beyotime, China) was utilized in accordance with the manufacturer’s instructions. Briefly, cells were washed by PBS for twice. The 4% paraformaldehyde was used to fix the cells for 15 min and then rinsed with distilled water for twice. After discarding the distilled water, BCIP/NBT substrate was incubated with cells for 40 min under a dark condition. The images were pictured and observed using an inverted microscope. Experiments were repeated in triplicate. An ALP assay kit (Beyotime, China) was used for the ALP activity assay. Briefly, RIPA lysis buffer containing phenylmethylsulfonyl fluoride (PMSF) was utilized to lyse BMSCs. Lysates were harvested from supernatants by centrifuging at 12,000 rpm for 10 min. The samples were incubated with reaction buffer for 15 min at 37 °C. After terminating the reaction, absorbance was detected at 520 nm and the concentration of protein was quantified via the BCA kit (Thermo Fisher Scientific, USA). Total protein concentration was used for normalization of ALP activity.

### Osteoclast differentiation and TRAP staining

BMDM were obtained from long bones of sacrificed C57BL/6 (6–8 weeks of age) mice. Cells were responded with the α-MEM containing 25 ng/ml M-CSF, 10% fetal bovine serum, 1% Penicillin–streptomycin for 24 h. Nonadherent cells in supernatants were harvested and cultured on 6-well plates with the medium supplemented with 50 ng/ml RANKL (R&D Systems, USA) and 25 ng/ml M-CSF (ρ = 2.0 × 10^5^ cells/cm^2^). The medium was changed every 2 d until mature osteoclasts had formed. TRAP staining kit (Sigma-Aldrich, USA) was utilized to stain the TRAP of osteoclasts via the following the manufacturers’ instructions. TRAP-positive cells with three or more nuclei were regarded as mature osteoclasts. The size and number of mature osteoclasts were observed and scored by an inverted microscope.

### cDNA synthesis and quantitative PCR analysis

RT-qPCR was used to quantify the gene expression levels. RNA was harvested through Trizol reagent (Invitrogen, USA) and converted into cDNA using PrimeScript RT Reagent kit (Takara, Janpan). And then RT-qPCR was performed using SYBR green (Takara, Janpan) by Bio-Rad CFX96 Real-Time PCR Detection System (Roche, Germany) with specific primers. β-actin was selected as loading control and the 2^−ΔΔCt^ method was performed to calculate relative gene expression. The experiment was repeated three times. Primer sequences used are listed in Additional file 1: Table S1.

### Western blot

The protein of cells or exosomes was isolated by RIPA lysis buffer, followed by centrifuging at 12,000 rpm for 10 min. After quantifying the concentration of protein via BCA kit, 40 μg proteins were separated on 10% SDS PAGE. After that, proteins were transferred onto a 0.5 μm pore size nitrocellulose membrane at 180 mA. Subsequently, the membranes were blocked by 5% skim milk in tris-buffered saline with Tween-20 (TBST) for 1 h and then incubated with primary antibodies for probing target protein at 4 ℃ overnight. The following day, the membranes were incubated with corresponding with secondary antibodies at room temperature for 1 h. The blots were detected by Odyssey Infrared Imager system (LI‐COR Bioscience, USA) according to the instructions. Antibodies covering GM130 (CST, USA), CD9 (Abcam, USA), TSG101 (Abcam, USA), and GAPDH (CST, USA) were prepared by TBST (1:1000). Secondary antibodies were prepared by TBST (1:2000).

### ELISA

The BMSCs or BMDM were co-cultured with M2-Exos for 24 h and then the medium was discarded and cells were rinsed with PBS for twice. Subsequently, fresh medium was added for an additional 24 h. Next, medium was harvested and centrifuged to remove the precipitate at 300 g for 10 min. ELISA kits (Lianke, China) were used to detect the expression of IL-10 from BMSCs or BMDM.

### Flow cytometric analysis

IL-10R on membranes was evaluated by Flow cytometry analysis. BMSCs or BMDM were collected and incubated with 1 μl of anti-IL-10R antibody (Biolegend, USA) in the dark for 30 min. Wash the samples sequentially with 1 ml wash solution for 3 times. The precipitate was obtained via centrifugation at 800 rpm and resuspended in 1 × PBS. Next, FITC-labeled secondary antibodies (Yeasen, China) was added for 30 min and the precipitate was collected via centrifugation at 800 rpm. Next, precipitate was resuspended in 300 µL PBS. Measurements were performed with Novocyte flow cytometer (ACEA) and data were analyzed with NovoExpress software.

### Murine periodontal disease model

All C57BL/6 (6–8 weeks of age) mice were obtained from the animal center of the Fourth Military Medical University and performed under protocols approved by the Animal Care and Use Committee. The mice underwent anesthesia by intraperitoneal injection. A 5–0 silk ligature was immersed in P. gingivalis bacterial solution (1 × 10^9^/CFU) for 1 h and then fixed to the maxillary left second molar of mice for 10 d throughout the duration of experiment to induce periodontitis. For exosomes treatment, 30 μl PBS or M2-Exos was injected locally when the ligature was removed. For antagomir treatment, the mixture of M2-Exos with anti-IL-10R antibody was injected locally after removing ligature.

### Micro-CT scanning and reconstruction

After intraperitoneal injection with overdose of pentobarbital sodium, maxillary alveolar bones were harvested from euthanized mice. The collected alveolar bones were fixed into 4% PFA before transferred into 75% ethanol. The further imaging examination was performed via the high-resolution micro-CT scanner (Yxlon, Germany). Three-dimensional reconstruction of each scan was done and reoriented to the same position for bone loss analysis with anatomic landmarks. The distance of six predetermined sites on both the buccal and palatal sides between the cementoenamel junction and the alveolar bone crest (CEJ-ABC distance) was measured as previously described [40].

### Histology and immunostaining analysis

Maxillae samples harvested from mice were fixed into 4% PFA for histological analysis. After that, the samples were demineralized and embedded in paraffin. Later on, the wax blocks were cut into 5-μm-thick sagittal sections which were stained with hematoxylin and eosin (H&E). An inverted microscope was used to captured histological images. For immunostaining analysis, the sections were blocked with 0.5% FBS at room temperature for 1 h. The sections were incubated with primary antibody of TRAP (Sevicepio, China) at 4 °C for 12 h, followed by secondary antibody at 37 °C for 1 h. Next, DAPI was used to satin sections at 37 °C for 10 min. Immunofluorescence signal was observed and captured by confocal microscope (Zeiss, Germany).

### Statistical analysis

The results were reported as mean ± SEM for at least three independent experiments. Data analyses in this experiment were performed by Student’s t-test between 2 groups, while one-way ANOVAs was used to examine differences among 3 groups by SPSS version 20.0 software. P < 0.05 was regarded as significance threshold (*, p < 0.05. **, p < 0.01. ***, p < 0.001).

## Supplementary Information

**Additional file 1: Fig. S1.** Identification of BMSCs. a Flow cytometry analysis of third‐generation BMSCs with antibodies including CD29-APC, CD45-PE and CD90-APC. b The adipogenic and osteogenic differentiation capability of BMSCs was detected by Oil Red o staining and Alizarin Red S Staining respectively. **Fig. S2.** Identification of BMDM. After 7 d of incubation with M-CSF, mature BMDM were defined as F4/80+CD11b+ cells by flow cytometry analysis. **Table S1.** Primer used in RT-qPCR.

## Data Availability

All data generated or analyzed during this study are included in this published article.

## Abbreviations

M2-Exos: Exosomes from reparative M2-like macrophages
BMSCs: Bone marrow stromal cells
BMDM: Bone marrow derived macrophage
OC: Osteoclast
IL-10: Interleukin-10
TRAP: Tartrate-resistant acid phosphatase
ALP: Alkaline phosphatase
Col1a: Type 1 collagen
Runx2: Runt-related transcription factor 2
Ocn: Osteocalcin
Acp: Acid phosphatase
Nfatc1: Nuclear factor of activated T-cells
c-Fos: Cellular oncogene
Mmp-9: Matrix metalloprotein 9
