# Supplementary material for: Exosomes derived from reparative M2-like macrophages prevent bone loss in murine periodontitis models via IL-10 mRNA
Source: J Nanobiotechnology. 2022 Mar 5;20:110. doi: 10.1186/s12951-022-01314-y (PMC8898524; doi:10.1186/s12951-022-01314-y)
Supplement: Supplementary file 1 — Additional file 1: Fig. S1. Identification of BMSCs. a Flow cytometry analysis of third‐generation BMSCs with antibodies including CD29-APC, CD45-PE and CD90-APC. b The adipogenic and osteogenic differentiation capability of BMSCs was detected by Oil Red o staining and Alizarin Red S Staining respectively. Fig. S2. Identification of BMDM. After 7 d of incubation with M-CSF, mature BMDM were defined as F4/80+CD11b+ cells by flow cytometry analysis. Table S1. Primer used in RT-qPCR. [file 12951_2022_1314_MOESM1_ESM.docx]

Additional **figures**


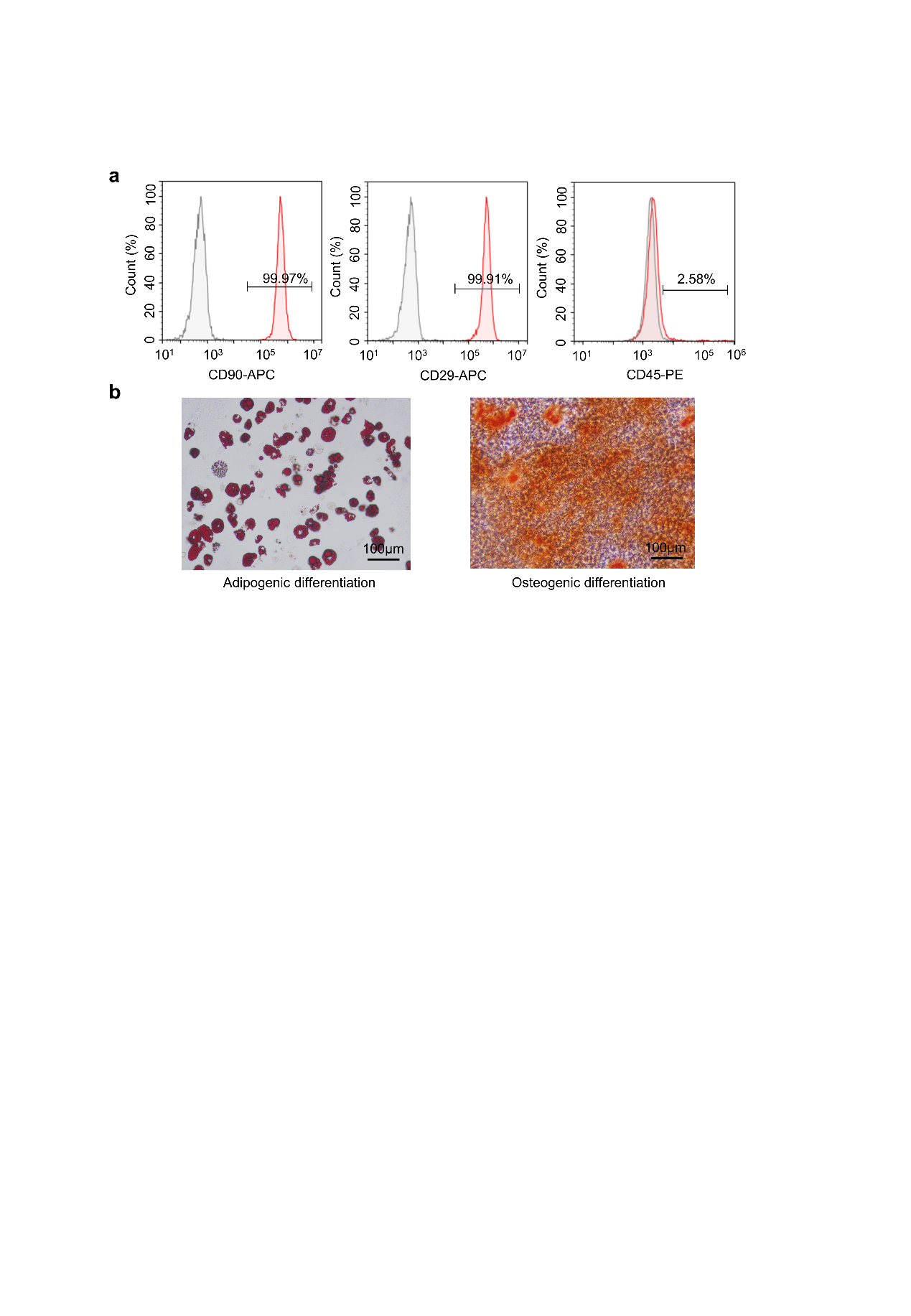


**Fig. S1 Identification of BMSCs.** a Flow cytometry analysis of third‐generation BMSCs with antibodies including CD29-APC, CD45-PE and CD90-APC. b The adipogenic and osteogenic differentiation capability of BMSCs was detected by Oil Red o staining and Alizarin Red S Staining respectively.


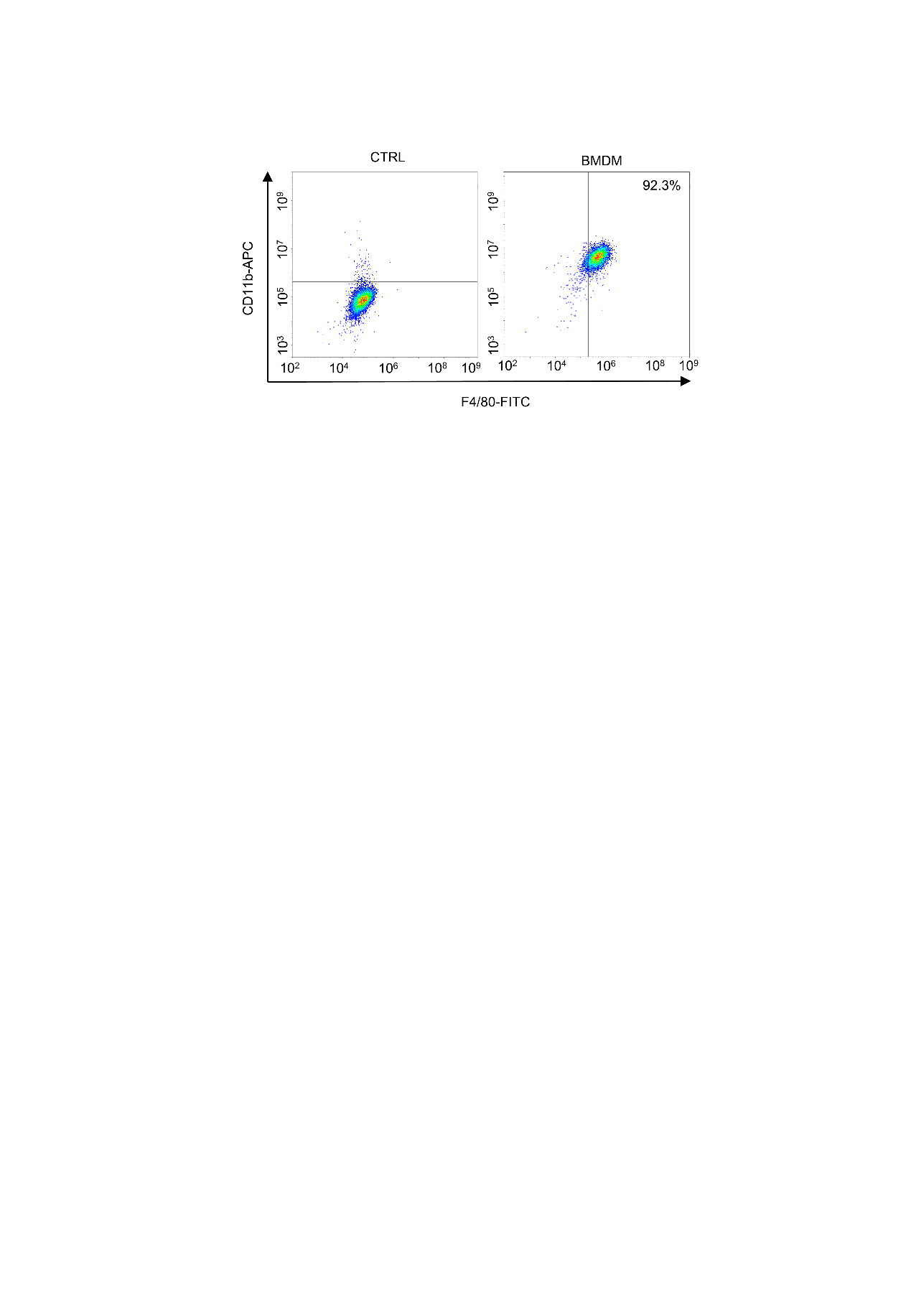


**Fig. S2 Identification of BMDM**. After 7 d of incubation with M-CSF, mature BMDM were defined as F4/80+CD11b+ cells by flow cytometry analysis.

**Additional Table**

**Table S1.** Primer used in RT-qPCR.

| Target | Sequence (*5*′-3′) |
| --- | --- |
| Acp | Forward: CCTCTGCAACTCTGGACTCTG  Reverse: AATCCATCTTGGCGGTGGG |
| Nfatc1 | Forward: GGGTCAGTGTGACCGAAGAT  Reverse: GGAAGTCAGAAGTGGGTGGA - |
| c-Fos | Forward: CCCGGCTTTCCCCAAACTT  Reverse: GCGCAAAAGTCCTGTGTGTT |
| Mmp-9 | Forward: GCCCTGGAACTCACACGACA  Reverse: TTGGAAACTCACACGCCAGAAG |
| Runx2 | Forward: AATTAACGCCAGTCGGAGCA  Reverse: CACTTCTCGGTCTGACGACG |
| Alp | Forward: TCCCCGCAACAGATCTCCTA  Reverse: AGGTGGAGTAGAGCCCTGAG |
| Ocn | Forward: TCTATGACCTGCAGAGGGCT  Reverse: ATAGCTCGTCACAAGCAGGG |
| Col1α | Forward: AGTGGTTTGGATGGTGCCAA  Reverse: GCACCATCATTTCCACGAGC |
| IL-10 | Forward: GCCTTATCGGAAATGATCCA  Reverse: AGGGGAGAAATCGATGACAG |
| β-actin | Forward: GGCTGTATTCCCCTCCATCG  Reverse: CCAGTTGGTAACAATGCCATGT |
